# Feedback at Test Can Reverse the Retrieval-Effort Effect

**DOI:** 10.3389/fpsyg.2019.01863

**Published:** 2019-08-13

**Authors:** Oliver Kliegl, Robert A. Bjork, Karl-Heinz T. Bäuml

**Affiliations:** ^1^Department of Psychology, University of California, Los Angeles, Los Angeles, CA, United States; ^2^Institute of Psychology, Universität Regensburg, Regensburg, Germany

**Keywords:** retrieval practice, feedback, retrieval effort, testing effect, episodic memory

## Abstract

The testing effect refers to the finding that retrieving previously encoded material typically improves subsequent recall performance more on a later test than does restudying that material. Storm et al. (2014) demonstrated, however, that when feedback is provided on such a later test the testing advantage then turns to a restudying advantage on subsequent tests. The goal of the present research was to examine whether there is a similar consequence of feedback when the difficulty of initial retrieval practice is modulated. Replicating prior research, we found that on an initial delayed test, recall of to-be-learned items was better following difficult than easy practice. Critically, however, providing immediate feedback on an initial delayed test reversed this pattern. Our findings are consistent with a distribution-based interpretation of how feedback at test modifies recall performance.

## Introduction

One of the major goals of education is to equip learners with knowledge that is both durable and flexible. We want such knowledge to remain accessible, even after long periods of disuse, and to transfer to the various contexts where it is relevant. A major challenge, however, is to determine—during the instruction process—whether those goals have been achieved. What an instructor has to work with, so to speak, is a learner’s *performance* during the instruction process, which decades of research has shown to be an unreliable guide to whether durable and flexible learning has been achieved (for a review, see Soderstrom and Bjork, 2015). Conditions of instruction that result in rapid improvements in performance can fail to support long-term retention and transfer, whereas other conditions—labeled *desirable difficulties* by Bjork (1994)—that pose challenges for learners and appear to slow the learning process can enhance long-term retention and transfer.

### Retrieval Practice as a Desirable Difficulty

Retrieval practice is one such desirable difficulty that has sprung to particular prominence within the last few years. A key finding about the benefits of retrieval practice is the so-called testing effect, which refers to the observation that active retrieval of some previously learned material can lead to better long-term retention than passive restudy of the material (e.g., Hogan and Kintsch, 1971; Whitten and Bjork, 1977; Roediger and Karpicke, 2006; for a review, see Roediger et al., 2011). In a typical testing effect task, participants are presented with the study material, for instance, a list of cue-target pairs (e.g., *pond* – FROG) and are subsequently either re-exposed to the study material (restudy condition), or are tested on it (*pond* – _____, retrieval-practice condition). When participants are later asked to recall the target information on a final test, they typically show a clear advantage in the retrieval-practice condition relative to the restudy condition.

Another key finding about retrieval practice is the retrieval-effort effect, which refers to the finding that more difficult retrieval practice is typically associated with better long-term retention (Bjork, 1975; Carpenter and DeLosh, 2006; Carpenter, 2009). Carpenter and DeLosh (2006), for instance, had participants study a list of items (e.g., *cabin*) and, in a subsequent practice phase, provided either only the first letter, or several letters as retrieval cues (e.g., *c___*, *ca___*, *cab__*, *cabi_*). Results showed that, on a subsequent free-recall test, fewer retrieval cues during practice led to better retention. That is, the greatest proportion of items retained on the free-recall test were those that were previously retrieved with a one-letter retrieval cue, and significantly fewer items were retained with two-letter, three-letter, or four-letter retrieval cues.

Both the testing-effect and retrieval-effort tasks show that more demanding practice conditions generally lead to better memory performance on a later criterion test than easier practice conditions. This pattern is all the more impressive when we consider the inherent disadvantage that is baked into more difficult practice conditions. Indeed, in testing-effect tasks, all of the study items are re-exposed—and thus repeated—during initial practice in the restudy condition, but participants will typically only be able to successfully retrieve—and thus repeat—a subset of the items in the more demanding retrieval-practice condition. Similarly, in retrieval-effort tasks, participants will typically be able to only retrieve a smaller proportion of items—and thus repeat those items—during more demanding than easier retrieval-practice tasks. Therefore, in both tasks, more difficult practice conditions lead to fewer items being repeated during practice (at least in the absence of feedback during practice) but still result in better long-term memory. We will later present a model that can account for this rather surprising regularity.

### The Testing Effect and Its Reversal

While the testing effect is very robust, and has been observed over a wide variety of study materials and experimental conditions (for a review, see Rowland, 2014), findings from a recent study by Storm et al. (2014) provide a striking demonstration of how the testing effect can be eliminated, or even reversed. Storm et al. (2014) employed a testing-effect task in which participants studied 36 Swahili-English pairs (e.g., *wingu* – CLOUD), and subsequently either retrieved or restudied subsets of that list, before participants were tested, 1 week later, on the English translations. Participants were given not only a single final test, but were given six final tests, on each of which they were presented with all the Swahili words from the initial study phase and asked to recall the English associate (*wingu* – ___). As expected, recall in the retrieval-practice condition was improved in a first final test (Test 1) relative to the restudy condition, replicating the standard testing-effect finding (e.g., Roediger and Karpicke, 2006).

Crucially, after each of the six final tests, participants received immediate feedback and were presented with the intact Swahili-English pair for restudy after each test trial (e.g., *wingu* – CLOUD). Providing such feedback had a massive impact on recall in the second final test (Test 2) as the testing effect was reversed, and recall performance was superior in the restudy relative to the retrieval-practice condition. On the following four final tests, performance continued to be higher in the restudy condition compared to the retrieval-practice condition (for a recent replication of these findings and possible electrophysiological markers of the effects, see Pastötter and Bäuml, 2016).

### A Distribution-Based Model of the Testing Effect and Its Reversal

**FIGURE 1:**
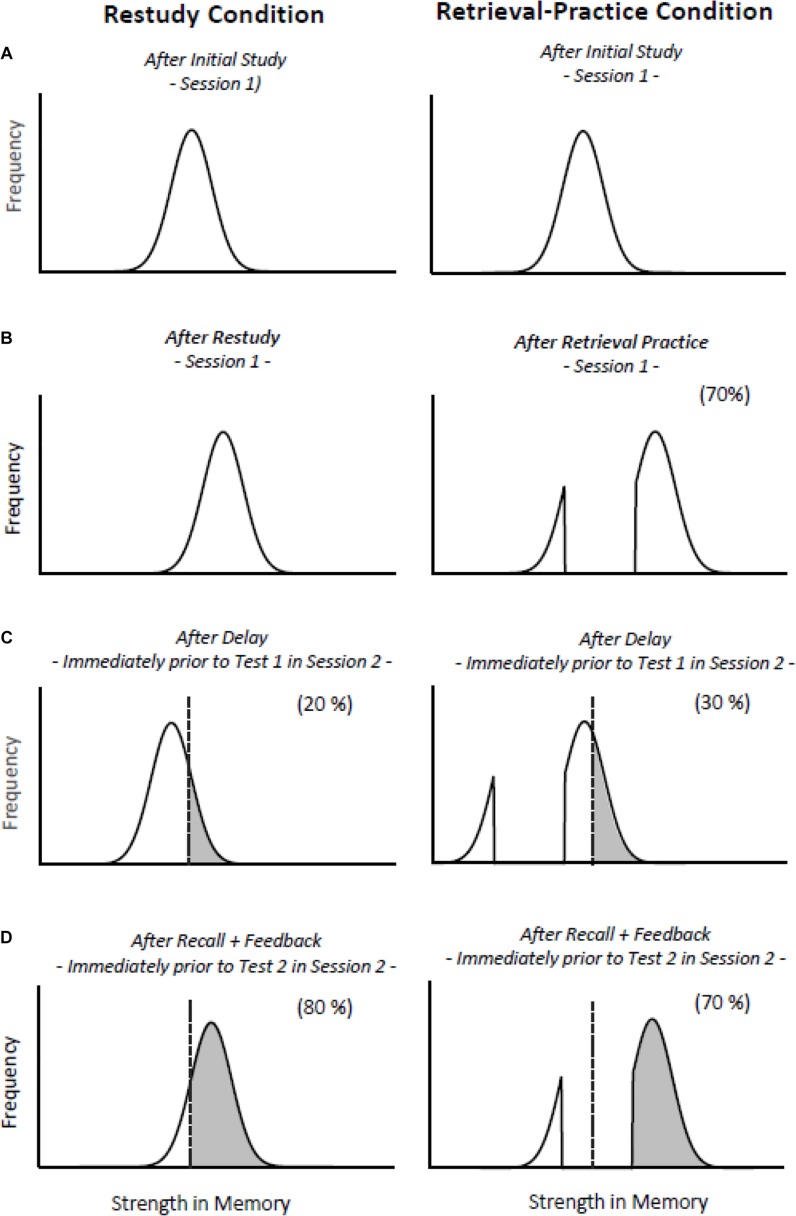
An illustration of the distribution-based bifurcation model (Halamish and Bjork, 2011; Kornell et al., 2011). The left and right columns show the hypothetical strength distributions of items after restudy practice and items after retrieval practice. **(A)** After initial study of a list of items in Session 1, the strength distributions are assumed to be identical and normal in the restudy and retrieval-practice conditions. **(B)** All restudied items gain memory strength about equally, whereas the distribution of the retrieval-practiced items becomes bifurcated: Successfully retrieved items get a boost in strength, whereas items not successfully retrieved do not gain any strength. Critically, the boost for successfully retrieved items is more pronounced than for restudied items. **(C)** All items are assumed to lose strength with delay, but, due to the bifurcated item distribution, the decline in recall may be less dramatic following retrieval practice than restudy. More of the retrieval-practiced than restudied items are above recall threshold after delay. The recall threshold is represented by the dotted, vertical line. **(D)** In both practice conditions, items that are successfully recalled in Test 1 are strengthened. Items that are not successfully recalled in Test 1 are strengthened by subsequent feedback, so that all items gain strength after recall and feedback.

Storm et al. (2014) explained their finding of a feedback-induced reversal effect on the basis of the recently proposed distribution-based model of the effects of retrieving versus restudying (Halamish and Bjork, 2011; Kornell et al., 2011). The first assumption of the model is that the to-be-learned items, after an initial study phase, end up—as a consequence of differences between items and moment-by-moment differences in learning efficiency—normally distributed along a memory strength continuum (see Figure 1A). The second assumption is that, during restudy, all items receive an incremental benefit in strength after each new practice trial, moving the item distribution of restudied items to the right. The third assumption is that, in the absence of corrective feedback, retrieval practice creates what Kornell et al. (2011) refer to as a “bifurcated” item distribution: Items that are successfully retrieved are strengthened to a higher degree than are corresponding restudied items, whereas items that are not successfully retrieved remain at their original memory-strength level (see Figure 1B). The fourth assumption of the model is that memory strengths of all items decrease with increasing retention intervals.

These assumptions are sufficient to explain the basic testing effect because, after a longer retention interval, many restudied items may fall below the recall threshold, whereas a larger proportion of the retrieval-practiced items may remain above threshold due to their high original strength level (see Figure 1C). Particularly important, the bifurcation model can also be extended to explain the reversed testing effect that arose in subsequent final tests of the Storm et al. (2014) study. To do so, in both practice conditions, it is assumed that (a) items that are successfully recalled in Test 1 show retrieval-induced strengthening and (b) items that are not successfully recalled in Test 1 are strengthened by subsequent feedback. In fact, there is evidence that items not successfully retrieved are subject to strengthening through feedback, whereas items successfully retrieved are hardly affected by feedback, if at all (Pashler et al., 2005; Kornell et al., 2011; Pastötter and Bäuml, 2016). Thus, all items, regardless of whether they were successfully retrieved or not, should gain strength after recall and feedback, so that more of the restudied than retrieval-practiced items should move beyond the recall threshold, resulting in a more pronounced increase in recall performance in the restudy than retrieval-practice condition, and a reversed testing effect in Test 2 (see Figure 1D).^1^

### The Present Study

The Storm et al. (2014) findings suggest that a single final recall test may not be sufficient to fully grasp the consequences of prior retrieval practice or prior restudying, because providing feedback through restudy on the first final test can reverse the testing effect. While this finding may have far-reaching implications for the status of retrieval practice as a desirable difficulty, before drawing any firm conclusions from the finding, it is important to investigate how general the effect is. To address the issue, we examined here whether feedback has analogous consequences for the retrieval-effort effect, that is, whether difficult retrieval practice leads to better recall on a first final test than easy retrieval practice—which is expected—but the effect reverses when on a second final test when there has been feedback on the first final test. To this end, we employed an experimental design similar to that employed by Storm et al. (2014). Participants first studied a list of weakly related cue-target word pairs (e.g., *disappear* – FADE, *jail* – CROOK), and on three later criterion tests were tested with immediate feedback. In each of these tests, participants were shown the cue word of each of the pairs and asked to type in the target word (e.g., *disappear* – ____, *jail* – ____). Test-1 recall levels were manipulated through degree of retrieval effort during a retrieval-practice task that followed the initial study phase. In this task, retrieval practice was relatively difficult or relatively easy, as participants were either presented with the cue word and the first two letters of the target word (e.g., *disappear* – FA___, easy practice) or the cue word and only the first letter of the target word (e.g., *jail* – C____, difficult practice). We expected that, relative to easy initial retrieval practice, difficult initial retrieval practice would result in a reduced recall performance during retrieval practice, but should lead to a better recall performance on Test 1 (Bjork, 1975; Carpenter and DeLosh, 2006).

**FIGURE 2:**
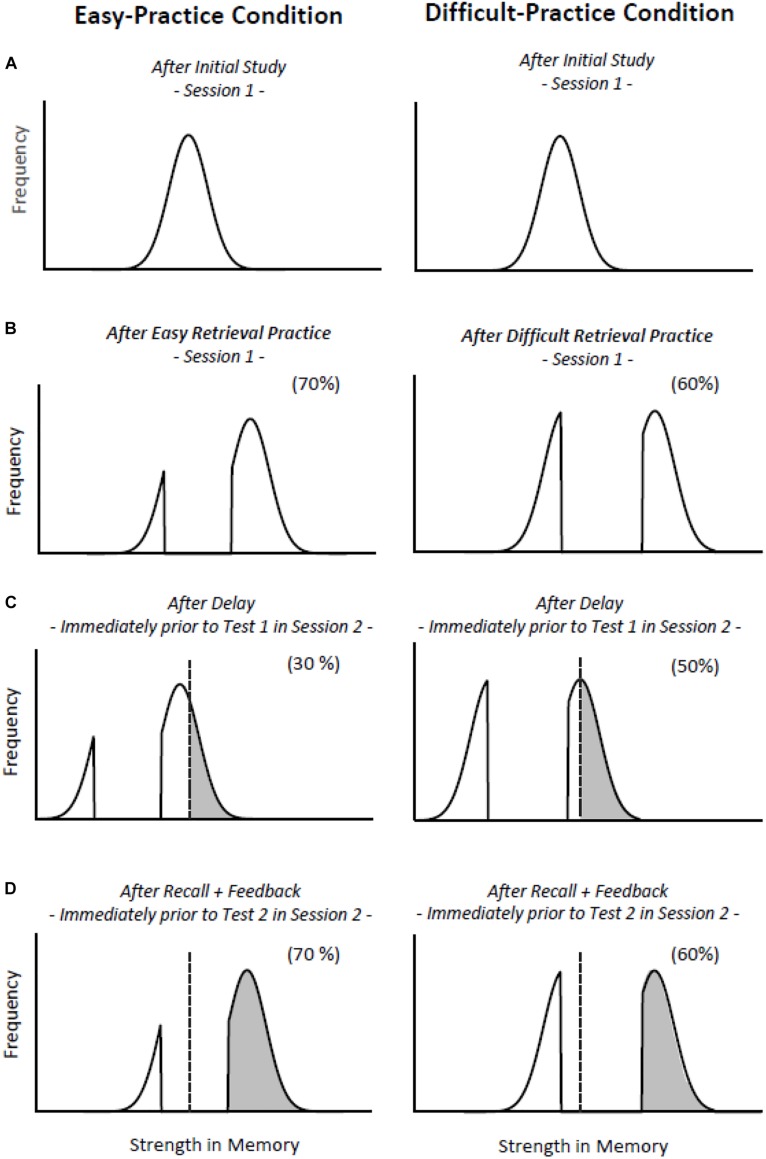
An application of the bifurcation model to the retrieval-effort effect. The left and right columns show the hypothetical strength distributions of items after easy and difficult retrieval practice. **(A)** After initial study, two identical strength distributions arise in the two retrieval practice conditions. **(B)** Successfully retrieved items get a boost in strength, whereas items not successfully retrieved do not gain any strength. This boost is more pronounced in the difficult-practice than the easy-practice condition. **(C)** All items lose strength with delay, but, due to the higher strength levels of the successfully retrieved items after difficult than easy retrieval practice, the decline in recall may be less dramatic in the difficult-practice than easy-practice condition, i.e., the retrieval-effort effect may arise in Test 1. **(D)** All items gain strength after recall and feedback, but because a higher ratio of items may be below the recall threshold in the easy-practice than difficult-practice condition, the increase in recall performance may be more pronounced in the easy-practice than the difficult-practice condition. Thus, in Test 2, the retrieval-effort effect may be reduced, or even reversed.

Critically, following Storm et al. (2014) we expected that after easy initial retrieval practice subsequent feedback would be more effective than after difficult retrieval practice. This expectation (which is counter-intuitive from most perspectives) follows from the bifurcation model, which assumes that successfully retrieved items get a stronger boost in the difficult-practice than easy-practice condition (compare Figures 2A,B, left vs. right panel). On Test 1, fewer items should fall above the recall threshold in the (higher-recall) difficult-practice than the (lower-recall) easy-practice condition (compare Figure 2C, left vs. right panel), which mimics the case in the (higher-recall) retrieval-practice condition and the (lower-recall) restudy condition (compare Figure 1C, left vs. right panel). Again, however, and mimicking the case in Storm et al. (2014) restudy and retrieval-practice conditions, the lower-recall conditions may benefit more from subsequent feedback than do the higher-recall conditions, because feedback may shift more items beyond the threshold in the lower-recall than in the higher-recall conditions (see Figure 2D). The effect observed in Test 1 may thus be reduced, or even reversed in subsequent tests.

## Materials and Methods

### Participants

Thirty-six students (30 females) at University of California, Los Angeles (UCLA) participated in this study. Participants’ mean age was 20.5 years (*SD* = 3.2 years), ranging from 18 to 38 years. Each participant was tested individually. Sample size was calculated using G^*^Power (Version 3.1.9.2; Faul et al., 2007). Based on prior work manipulating difficulty of retrieval practice (e.g., Carpenter, 2009; Pyc and Rawson, 2009), we assumed a medium-sized retrieval-difficulty effect (*d* = 0.5). Type-1 error probability was set to 0.05, power was set to 0.80. Based on these input variables, G^*^Power suggested a sample size of 34. Due to organizational reasons, the actual number of tested individuals turned out slightly higher (i.e., 36), but we chose not to remove any participants from the final sample.

### Material

The stimuli were 36 weakly related cue-target pairs from the Jacoby (1996) norms. The association frequency of the competitive fragment completions ranged from 0.03 to 0.59 (*M* = 0.27). The assignment of word pairs to practice conditions was randomized across participants.

### Design and Procedure

The experiment consisted of two sessions, with a delay of 7 days between them. Session 1 consisted of an initial study phase and a practice phase. In the initial study phase, participants studied 36 weakly related word pairs (e.g., *disappear* – FADE, *jail* – CROOK). Participants were told that, on a later retention test, they would be presented with the left word of each pair (i.e., the cue word) and be asked to produce the right word of each pair (i.e., the target word). Word pairs were presented one at a time in the middle of a computer screen for 5 s each.

After the study phase, participants played Tetris for 60 s before they engaged in two blocks of randomly intermixed easy and difficult retrieval practice trials. That is, retrieval effort (easy practice vs. difficult practice) was manipulated within participants. In particular, participants were either presented with the cue word and the first two letters of the target word (e.g., *disappear* – FA___, easy practice) or the cue word and the first letter of the target word (e.g., *jail* – C___, difficult practice). In both conditions, the cue word and fragment were shown for 5 s, and participants were asked to type in the entire target word while the cue word and fragment were present on the screen. No feedback was provided. The assignment of word pairs to practice conditions was randomized across participants but was maintained across blocks. No feedback was provided during the initial retrieval-practice task.

Participants returned to the lab 7 days later for Session 2. They were informed that they would be tested on all previously studied items and that each test trial would be followed by feedback in which the corresponding word pair would be re-presented intact. In Test 1, the 36 cue words were presented at the center of the computer screen, one at a time. For each cue, participants had 5 s to type in the corresponding target word. Order of cues was randomized. Immediate feedback was provided by showing the corresponding word pair intact for 2 s. Test 1 was immediately followed by Test 2, which was immediately followed by Test 3. The setup of Tests 2 and 3 was identical to Test 1, in that participants were shown each of the 36 cue words for 5 s per item, and feedback was provided after each trial for 2 s.

## Results

### Retrieval Success in the Practice Phase (Session 1)

**FIGURE 3:**
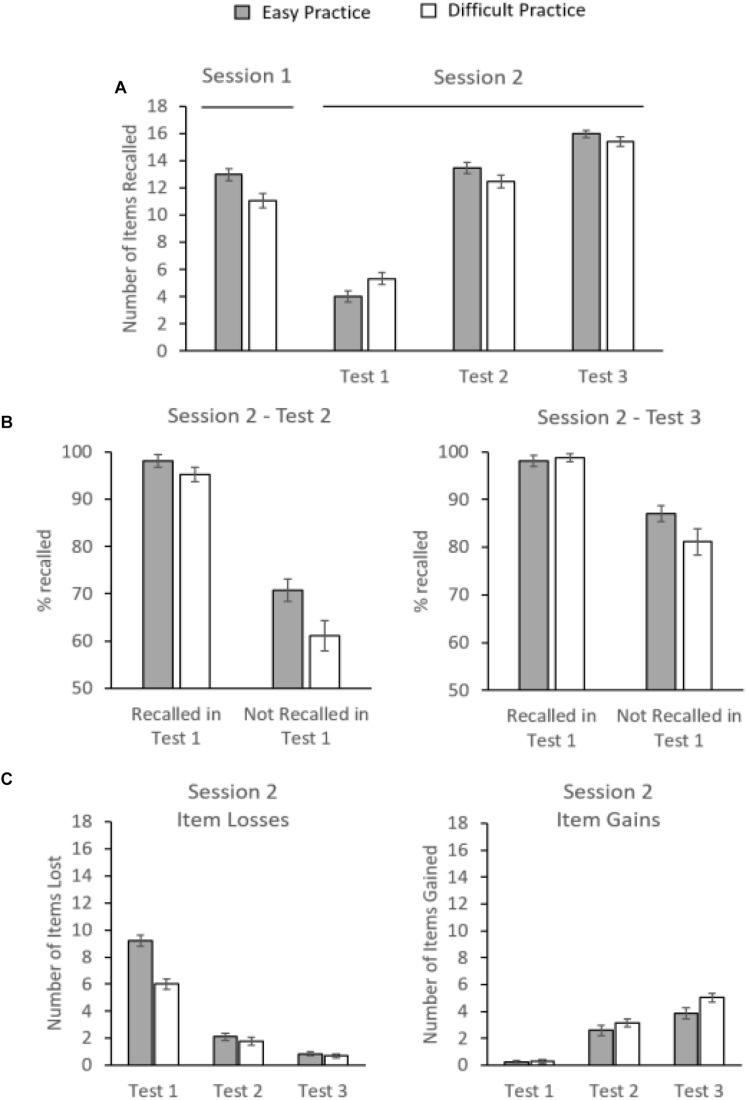
**(A)** Session 1: Mean retrieval success during the initial retrieval-practice cycle in Session 1. Session 2: Percentage of correctly recalled study items in Session 2 as a function of PRACTICE TYPE (difficult practice, easy practice) and TEST (Test 1, Test 2, and Test 3). **(B)** Left panel: Conditional analyses of recall in Test 2 showed that the reversed retrieval-effort effect arose from a practice effect for items that were *not* recalled in Test 1. No such practice effect arose for items that were successfully recalled in Test 1. Right panel: Conditional analyses of recall in Test 3 showed that the reversed retrieval-effort effect arose from a practice effect for items that were *not* recalled in Test 1. No such practice effect arose for items that were successfully recalled in Test 1. **(C)** Left panel: Item losses on Tests 1, 2, and 3 relative to initial retrieval practice. Right panel: Item gains on Tests 1, 2, and 3 relative to initial retrieval practice. Error bars represent standard errors.

As expected, participants’ mean recall performance across the two initial retrieval-practice cycles in Session 1 was significantly higher in the easy-practice than difficult-practice condition (13.2 items vs. 11.2 items), *t*(35) = 4.198, *p* < 0.001, *d* = 0.715 (see Figure 3A).

### Recall Performance During the Test Phase (Session 2)

Figure 3A also shows recall performance during the final-test phase as function of PRACTICE (easy, difficult) and TEST (Test 1, Test 2, and Test 3). A 2 × 3 ANOVA revealed no main effect of PRACTICE, *F*(1,35) < 1, but a main effect of TEST, *F*(2,70) = 626.690, *MSE* = 3.771, *p* < 0.001, partial η^2^ = 0.95, reflecting that, overall, recall was higher in Tests 2 and 3 than Test 1. There was also a significant interaction between the two factors, *F*(2,70) = 25.313, *MSE* = 1.092, *p* < 0.001, partial η^2^ = 0.42, suggesting that type of practice had differential effects on recall performance across the three tests. Regarding Test 1, planned comparisons showed that participants’ mean recall in the difficult-practice condition was better than in the easy-practice condition (5.3 items vs. 4.0 items), *t*(35) = 4.739, *p* < 0.001, *d* = 0.795, thus replicating the retrieval-effort effect (e.g., Carpenter and DeLosh, 2006). Crucially, however, this pattern was reversed in subsequent tests, as performance in the easy-practice condition was superior to the difficult-practice condition, in both Test 2 (12.4 items vs. 13.5 items), *t*(35) = −3.164, *p* = 0.003, *d* = −0.537, and Test 3 (15.4 items vs. 16.0 items), *t*(35) = −2.127, *p* = 0.041, *d* = −0.377. There was no significant interaction between PRACTICE and TEST from Test 2 to Test 3, *F*(1,70) < 1, although both PRACTICE and TEST influenced recall performance, *all ps* < 0.005.

Conditional recall analyses further showed that for those items that were *not* recalled in Test 1 the reversal in Test 2 arose from an easy-practice over difficult-practice benefit (easy practice: *M* = 70.7%, *SE* = 2.4%; difficult practice: *M* = 61.1%, *SE* = 3.2%), *t*(34) = 3.897, *p* < 0.001, *d* = 0.669, and also the reversal in Test 3 arose from an easy-practice over difficult-practice benefit (easy practice: *M* = 87.0%, *SE* = 1.7%; difficult practice: *M* = 81.1%, *SE* = 2.7%), *t*(34) = 2.859, *p* = 0.007, *d* = 0.484. For items that were successfully recalled in Test 1, no such effect of practice condition arose in either Test 2 (easy practice: *M* = 98.1%, *SE* = 1.3%; difficult practice: *M* = 95.3%, *SE* = 1.5%), *t*(34) = 1.89, *p* = 0.067, *d* = 0.310, or Test 3 (easy practice: *M* = 98.1%, *SE* = 1.1%; difficult practice: *M* = 98.8%, *SE* = 0.9%), *t*(34) < 1 (see Figure 3B).^2^

### Item Gains and Item Losses

We next sought to determine (i) how many of the items that were recoverable in Session 1 were unrecoverable in Session 2, and (ii) how many items that were unrecoverable in Session 1 were recoverable in Session 2. To this end, we analyzed items losses and item gains across the three tests in Session 2 relative to initial retrieval practice in Session 1 (see Figure 3C). Losses on Test 1 were items reported during retrieval practice but not on Test 1, losses on Test 2 were items reported during retrieval practice but not on Test 2, and losses on Test 3 were items reported during retrieval practice but not on Test 3. Likewise, gains on Test 1 were studied items reported on Test 1 but not during retrieval practice, gains on Test 2 were items reported on Test 2 but not during retrieval practice, etc.

Regarding item losses, a 2 × 3 ANOVA with the within-subjects factors of PRACTICE (easy, difficult) and TEST (Test 1, Test 2, and Test 3) revealed a significant main effect of PRACTICE, *F*(1,35) = 17.308, *MSE* = 4.732, *p* < 0.001, partial η^2^ = 0.331, reflecting that item losses were more pronounced in the easy-practice than difficult-practice condition, and a main effect of TEST, *F*(2,70) = 437.300, *MSE* = 2.190, *p* < 0.001, partial η^2^ = 0.93, reflecting that item losses decreased from Test 1 to Test 2 to Test 3. There was also an interaction between factors, *F*(2,70) = 45.223, *MSE* = 1.153, *p* < 0.001, partial η^2^ = 0.56, suggesting that type of practice had differential effects on item losses across the three tests. Indeed, planned comparisons showed a reliable difference between the difficult-practice and the easy-practice condition only for Test 1 (6.0 items vs. 9.2 items), *t*(35) = −6.551, *p* < 0.001, *d* = −1.093, but not for Test 2 (1.8 items vs. 2.1 items), *t*(35) < 1, and Test 3 (0.7 items vs. 0.9 items), *t*(35) < 1.

Regarding item gains, the same ANOVA revealed a significant main effect of PRACTICE, *F*(1,35) = 4.381, *MSE* = 4.194, *p* = 0.044, partial η^2^ = 0.11, reflecting that item gains were more pronounced in the difficult-practice than easy-practice condition, and a main effect of TEST, *F*(2,70) = 134.657, *MSE* = 2.387, *p* < 0.001, partial η^2^ = 0.79, reflecting that item gains increased from Test 1 to Test 2 to Test 3. There was also an interaction between factors, *F*(2,70) = 4.779, *MSE* = 1.223, *p* = 0.011, partial η^2^ = 0.12, suggesting that type of practice had differential effects on item gains across the three tests. Indeed, planned comparisons found a reliable difference between the difficult-practice and easy-practice condition only for Test 3 (5.0 items vs. 3.9 items), *t*(35) = 2.701, *p* = 0.011, *d* = 0.459, but not for Test 1 (0.3 items vs. 0.3 items), *t*(35) < 1, and Test 2 (3.2 items vs. 2.6 items), *t*(35) = 1.682, *p* = 0.102, *d* = 0.231.

## Discussion

Results showed that, on the two retrieval-practice cycles of Session 1, mean success rates were higher in the easy-practice than difficult-practice condition, suggesting that the manipulation of retrieval difficulty was successful. A first delayed criterion test after 1 week (Test 1) then showed that recall performance was enhanced in the difficult-practice condition, relative to the easy-practice condition, reflecting the typical retrieval-effort effect (e.g., Whitten and Bjork, 1977; Carpenter and DeLosh, 2006). Crucially, however, the retrieval-effort effect was reversed on the subsequent Tests 2 and 3, as recall performance in the easy-practice condition was now superior to the difficult-practice condition. These results extend the Storm et al. (2014) findings by demonstrating that feedback on a delayed criterion test can not only reverse the testing effect, but can also reverse the retrieval-effort effect. Results from the conditional recall analyses further showed that the reversal of the retrieval-effort effect in Tests 2 and 3 arose from a easy-practice over difficult-practice benefit only for those items that were not recalled in Test 1. In fact, in Tests 2 and 3, no effect of practice condition arose for items that were successfully recalled in Test 1.

### Relation to the Bifurcation Model

The observed retrieval-effort effect on Test 1 is consistent with the assumption of the bifurcation model that, after a longer delay (and immediately prior to Test 1), more items should fall below the recall threshold in the easy-practice condition than in the difficult-practice condition. The bifurcation model can also explain the reversed retrieval-effort effect that was observed on Tests 2 and 3 because the model assumes that more items are in close proximity to the recall threshold in the easy-practice than the difficult-practice condition before feedback is provided on Test 1. Therefore, a higher number of items should pass the recall threshold after feedback in the easy-practice than difficult-practice condition (compare Figures 2C,D), and become recallable, which is what the present results demonstrate.

In particular, the model assumes that the items from the easy-practice conditions which are not recalled in Test 1 generate the reversal of the retrieval-effort effect in Tests 2 and 3. This is because, relative to the difficult-practice condition, more of the items that were recovered during initial practice should be in close proximity to the recall threshold before feedback is provided in Test 1 in the easy-practice condition, which makes it likely that feedback moves them over the recall threshold and thus makes them recallable in Test 2. In contrast, the items that are successfully recalled in Test 1 should not contribute to the reversal. The present results from the conditional recall analyses are consistent with these assumptions.

The present findings on item losses complement the findings from the conditional analyses and provide further support for the distribution-based bifurcation model. While we found that, relative to initial practice, there were substantial item losses in Test 1 in the easy-practice and difficult-practice conditions, the reduction in item losses in Test 2 (after feedback) was more pronounced in the easy-practice than difficult-practice condition. These findings are also well in line with the model’s assumption that, in the easy-practice condition, feedback induces more items to be shifted across the recall threshold than in the difficult-practice condition, because this should reduce item losses more in the easy-practice than difficult-practice condition.

While the bifurcation model can handle the loss findings of the present experiments in a relatively straightforward way, it cannot easily account for the present finding that item gains in both the lower-and higher-recall conditions were somewhat increased after feedback (i.e., on Tests 2 and 3). Indeed, as pointed out earlier, the model predicts that feedback should shift the items that were successfully retrieved during initial retrieval practice beyond recall threshold, thus resulting in reduced item losses, but should hardly shift items that were not retrieved during initial retrieval practice beyond threshold (compare right panels of Figures 1C,D). Different variants of the model may account for the gain findings, for instance, a variant that allows for a random fluctuation of the recall-threshold position on the memory strength dimension on each single test trial, or a random fluctuation of the items’ memory strength *per se*. Examining such variants in more detail is a high priority for future research.

### Relation to Prior Work

Storm et al. (2014) showed that feedback on an initial delayed test can be more beneficial for initially restudied than for initially retrieval-practiced items, thus reversing the testing effect on subsequent tests. Pastötter and Bäuml (2016) replicated and extended Storm et al.’s (2014) finding by showing that the reversal effect on Test 2 is generated primarily by those restudied items that are *not* recalled on Test 1. The present experiment generalizes both of these lines of findings to the retrieval-effort effect, by demonstrating that feedback at test can be more beneficial for easier, compared to more demanding, retrieval-practice conditions. This regularity may, however, only apply when no feedback is provided during initial retrieval practice. Indeed, in a second experiment, Storm et al. (2014) found that when they provided feedback via restudy during both initial retrieval practice and on the delayed test, there was not only an intact testing effect on the first delayed test but, critically, also on subsequent tests. This absence of a reversed testing effect following feedback at test may have arisen because feedback during retrieval practice prevented a bifurcation of the item strength distributions in the first place (Kornell et al., 2011). While the present study did not include an experimental situation in which feedback during initial retrieval practice and on the later test is provided, it appears likely on the basis of the Storm et al. (2014) findings that no reversed retrieval-effort effect would arise under such circumstances. Future research may address the issue in more detail.

Investigating possible neural markers of the feedback effect, Pastötter and Bäuml (2016) recorded participants’ electroencephalogram (EEG) during feedback. They found feedback-related effects in the alpha/lower-beta frequency range (12–16 Hz) and in the slow theta frequency range (2–4 Hz). Critically, both effects were independent of practice condition. Results further showed that feedback only strengthened those items that were *not* recalled in Test 1, but not the items that *were* recalled in Test 1. Both these findings are consistent with the bifurcation model if we assume that feedback via restudy strengthens all items below threshold to the same degree, regardless of whether they were retrieved or restudied in Session 1, but leaves the items above threshold unaffected. Future studies may examine whether a similar equivalence in feedback effects arises when comparing feedback effects after difficult vs. easy retrieval practice. If so, such findings would indicate that feedback effects depend primarily on whether items were successfully recalled or not (Pashler et al., 2005; Kornell et al., 2011; Pastötter and Bäuml, 2016), and much less, if at all, on the nature of the factors that initially reduced the recall performance. Corresponding results would provide new insights into the effects of feedback and impose important restrictions on theories of feedback effects.

Finally, it is worth considering results from prior work indicating that motivational factors can affect how feedback is processed. For instance, recent research suggests that when participants are unable to provide a correct answer at test, their motivation to learn the correct answer when it is subsequently presented as feedback may be particularly high (e.g., Kang et al., 2009; Potts and Shanks, 2019). Our participants may thus have allocated more attention to a feedback opportunity after they were unable to recall the target word than when they were able to do so. However, it is less obvious how motivational factors would explain the more pronounced increases in recall performance from Test 1 to Test 2 (and from Test 1 to Test 3) that were observed in the easy-practice condition, relative to the difficult-practice condition. To account for the reversal effect on the basis of a motivational account, we would have to assume that participants in the easy-practice condition were allocating systematically more attentional resources to feedback than in the difficult-practice condition. This seems rather unlikely because, in our experiment, easy-practice and difficult-practice trials were randomly intermixed for each participant. As a result, on most test trials, participants were probably not even aware whether a particular item was originally practiced in the easy-practice or difficult-practice condition 1 week before. It therefore seems rather unlikely that allocation of attentional resources differed dramatically during feedback processing for the two practice conditions.

### Possible Boundary Conditions of the Differential Feedback Effects

The present results together with the results from the prior work by Storm et al. (2014) and Pastötter and Bäuml (2016) converge on the view that feedback at test is more beneficial in retrieval situations that initially (i.e., on a first retention test) result in inferior recall performance, relative to retrieval situations that initially result in higher recall performance. While indeed such pattern may occur quite frequently, exceptions should arise. For instance, the bifurcation model suggests that the reversal of the retrieval-effort effect may no longer arise if the recall test is delayed much longer than a single week. In such case, the strength distributions of the successfully retrieved items should be considerably further to the left than in Figure 2C, so that most of the successfully retrieved items are below the recall threshold in both practice conditions. The increase in delay would reduce recall rates but might still show a retrieval-effort effect in Test 1, with more items being recalled in the difficult- than easy-practice condition. If feedback was then provided during Test 1, a higher number of the items may get shifted past the recall threshold in the difficult-practice than easy-practice condition, thus leading to an even more pronounced retrieval-effort effect in Test 2 than Test 1. Analogous scenarios can be created for the standard testing effect, meaning that there should be circumstances (like for very long delay) where feedback at test may magnify, instead of reduce, the effects.

Such a pattern has recently been reported in two studies which examined the influence of sleep on the testing effect. In the one of the two studies, it was shown that a 12-hour delay interval filled with sleep can eliminate the testing effect (Bäuml et al., 2014). In the other study, the finding was replicated and it was demonstrated that this influence of sleep disappears for prolonged delays of 24 h or 7 days (Abel et al., 2019). On the basis of the assumption that, similar to feedback, sleep strengthens all types of memories to a similar degree, these findings are well in line with the distribution model. Indeed, after a delay of 12 h, sleep should mostly shift restudied items beyond recall threshold, whereas retrieved items are already too far above recall threshold to show any additional sleep benefit after a 12-hour delay, thus resulting in the observed reduction of the testing effect. With prolonged delay, like 7 days, however, most of the study items should cross below threshold in both practice conditions, thereby unmasking potential additional benefits of sleep-associated strengthening. Just like prolonged delay can reduce the modulating role of sleep for the testing effect, it may also reduce the modulating role of feedback for the testing effect.

## Conclusion

The results of the present experiment extend and generalize prior work that showed feedback to reverse the testing effect, by showing that feedback at test can also reverse the retrieval-effort effect. Both of these reversal effects can be explained by the bifurcation model. From a more applied perspective, the findings generally support the view that retrieval practice can serve as a desirable difficulty, with more demanding retrieval-practice conditions resulting in better long-term retention than easier (retrieval-) practice conditions. This regularity, however, only applies to learning situation in which *no* feedback via restudy is provided during a delayed retention test. In the presence of such feedback—and in the absence of feedback during initial retrieval practice—the beneficial effects of more demanding practice may be eliminated, or even reversed.

## Data Availability

All data can be found at Open Science Framework, https://osf.io/ft5xw/.

## Ethics Statement

This study was carried out in accordance with the recommendations of the UCLA Institutional Review Boards with written informed consent from all subjects. All subjects gave written informed consent in accordance with the Declaration of Helsinki. The protocol was approved by the UCLA Institutional Review Boards.

## Author Contributions

OK developed the study concept and experimental design, performed the data analysis, and drafted the manuscript. RB and K-HB helped developing the study concept and also gave critical input for various revisions of the manuscript. All authors approved the final version of the manuscript for submission.

## Conflict of Interest Statement

The authors declare that the research was conducted in the absence of any commercial or financial relationships that could be construed as a potential conflict of interest.

## Footnotes

1 Figure 1D depicts an idealized situation, in which the retrieval-related and feedback-related gains in memory strength that occur during Test 1 are assumed to be equal. The expectations derived from the bifurcation model in the present study, however, do not depend on this assumption and would not change if retrieval-induced and feedback-induced strengthening differed somewhat in degree.

2 A single participant who did not recall any items in Test 1 in the easy-practice condition was not included in the conditional analyses. This was done because division by zero is not defined, and therefore, no conditional probabilities could be calculated for this participant.
